# Reconsidering the role of language in medicine

**DOI:** 10.1186/s13010-018-0058-z

**Published:** 2018-06-05

**Authors:** Berkeley Franz, John W. Murphy

**Affiliations:** 1 Department of Social Medicine, Heritage College of Osteopathic Medicine, Ohio University, Grosvenor311, Athens, OH USA; 2 Department of Sociology, University of Miami, Merrick 121 E, Coral Gables, FL USA

**Keywords:** Language, Philosophy of medicine, Narrative medicine, Doctor-patient communication

## Abstract

**Background:**

Despite an expansive literature on communication in medicine, the role of language is dealt with mostly indirectly. Recently, narrative medicine has emerged as a strategy to improve doctor-patient communication and integrate patient perspectives. However, even in this field which is predicated on language use, scholars have not specifically reflected on how language functions in medicine.

**Methods:**

In this theoretical paper, the authors consider how different models of language use, which have been proposed in the philosophical literature, might be applied to communication in medicine. In particular, the authors contrast the traditional, indexical thesis of language with new models that focus on interpretation instead of standardization.

**Results:**

The authors demonstrate how paying close attention to the role of language in medicine provides a philosophical foundation for supporting recent changes in doctor-patient communication. In particular, interpretive models are at the foundation of new approaches such as narrative medicine, that emphasize listening to patient stories, rather than merely collecting information.

**Conclusion:**

Ultimately, debates regarding the role of language which have largely resided in non-medical literatures, have important implications for describing communication in medicine. In particular, interpretive models of language use provide an important rationale for facilitating a more robust dialogue between doctors and patients.

## Background

In the field of medicine discussions of language have been relatively rare. Many readers, at first, may object to this claim. After all, many articles and books examine the communicative competence of clinicians [1, 2] Medical students, for example, are constantly reminded that they must learn to interact effectively with patients. Additionally, issues related to translation, interpretation, and cultural competence are constantly discussed [3, 4].

Perhaps a better way to state the problem is that in medicine language is dealt with mostly indirectly. Many studies focus, for example, on the physician-patient relationship, the need for dialogue, the proper interpretation of clinical records, and the power dynamic that exists between doctors and their patients [5–7]. In other words, the quality and type of interaction that occurs in clinical settings are often the focus of studies in the literature on medical encounters.

Most recently, narrative medicine has emerged as a prominent technique for integrating language and literature into medicine [8–10]. The main point of this approach is that patients construct stories about their lives, including their illnesses, that are vital to understanding their social and bodily conditions. The claim made by narrative medicine is that physicians, due to their training and focus on physical elements, have historically ignored these storylines [11]. As a result, physicians make diagnoses or design interventions that are culturally insensitive or, at times, irrelevant. As a corrective, the framework of narrative medicine encourages physicians to truly listen to their patients.

Given this research and recent trends, how can a legitimate claim be made that language is ignored in medicine? The answer is that in each of these cases, including narrative medicine, language is involved but is not the primary focus of attention. Any explanation of the interaction patterns of patients and physicians presupposes the use of language, but the nature of language itself is not usually in question. Even in most treatments of narrative medicine, the stories patients tell are presumed to be informative. The issue remains to be explored, however, whether the nature of language contributes to this insight. The aim of this paper, therefore, is to demonstrate why focusing on divergent theories of language is a practical consideration for clinicians. Specifically, we argue that moving beyond the traditional theory of language is necessary in order to take seriously the stories offered by patients in the clinical encounter.

Critical to narrative medicine is that both patients and clinicians bring perspectives to the clinical encounter that are relevant to patient care. Any concern for improving communication, so that these stories can be explored and better guide a care plan, is only logical. When persons speak, for example, they expect others to listen, and both physicians and patients are no exception. From the perspective of patients, having physicians listen carefully to their insights is often appreciated given the time constraints of clinical practice. Evidence suggests that patients feel comfortable with caregivers who are attentive, and believe that such care is effective [12].

In this regard, narrative medicine deals with metaphors, literary anecdotes, and the need to approach persons or clinical records openly. Additionally, empathy is extolled but this skill presupposes language use [13]. In these cases, the nature of language is not the prime concern. Instead scholars of narrative medicine emphasize sensitivity to the narratives that both clinicians and patients bring to the clinical meeting. These stories are believed to supply details that are often overlooked, but which are important to quality care.

But is there a more compelling reason for physicians and other service providers to pay attention to language? The guiding theme of this paper is that the nature of language provides such a reason, and therefore the fundamental character of speech should be investigated. Philosophers and some other scholars have been raising this issue for some time, but in medicine such an inquiry has been mostly missing [14].

The defining methodology of this discussion is phenomenology, particularly the anti-dualistic stance of this philosophy. When Husserl states that “consciousness is always conscious of something,” he is undermining a long-standing position that diminished the influence of language and encouraged the pursuit of objectivity [15]. But with language thoroughly intertwined with whatever is known, interpretation is elevated in importance by Husserl and others who subsequently emphasized the importance of interpretation. According to these proponents, events or behavior must be interpreted, or contextualized, properly to be treated as facts or evidence [16]. The influence of language, in other words, is unavoidable and provides the entrée to a patient’s world that is necessary for a relevant portrayal of illness or health.

The key issue here is that clinicians should be concerned about language use, given that interpretation is pervasive and plays a primary role in communication. For these reasons, a wide range of health professionals should be interested in language. But in the field of medicine, discussions of communication have mostly focused on the transmission or dissemination of information [17]. Nonetheless, there is something fundamental about language that demands the attention of clinicians. Perhaps language offers access to information that is vitally important to proper care.

### Traditional role of language

In traditional portrayals, language is described as being a tool [18]. This apparatus, moreover, differentiates humans from animals, and represents a huge advance in evolutionary development. With the help of language, humans are able to identify specific elements, such as behaviors and events, and make particular differentiations. Everyday life, according to this scenario, is no longer murky but, through socialization and training, can be clearly demarcated.

The indexical thesis is the outgrowth of describing language as a tool. There are two parts to this position. The first is that language is tied to cognition and represents human expressions. The second is that these expressions are attached to reality. This attachment, however, is crucial. Specifically, the link that exists serves to distinguish factors and make these elements known.

The metaphor that proponents often introduce at this juncture is of language as a pointer. As sentiments or expressions are attached to objects, language is able to identify things, highlight differences, distinguish background from foreground, illustrate context, and isolate key features. Simply put, language can make distinctions and discriminations and impose order on an otherwise nebulous mass of input. What is important are the connections present between language and the referents that are identified.

The key benefit of this rendition of language is that clarity can be established. In order to achieve this aim, however, a precise link has to exist between a linguistic expression and a particular referent. Language and referents must be clearly, unambiguously aligned. When these connections are clear, understanding is possible. Within the framework of the indexical thesis, precision is highly valued. As much noise, or ambiguity, must be eliminated as possible from the relationship between language and all referents. Language use, accordingly, is assumed to be clear when the referent of an expression is precisely identified.

When a person says “house,” for example, a particular phenomenon should come to mind, with all of the pertinent characteristics. At first, the link between language and this referent may not seem problematic. In the context of medicine, on the other hand, these linkages are not so obvious. When patients say that they do not feel well or are experiencing pain, establishing clarity is not always easy. A certain amount of interpretation is, therefore, inevitable [19].

Clinicians, nonetheless, must try to clarify the connection between an expression of pain and a physical referent. This association, however, is mired in a host of social and cultural considerations that make this identification difficult. In some cases of chronic pain, these associations are incredibly vague and thus dismissed as fantasy or as medication-seeking behavior [20, 21]. What weighs heavily in this decision is the degree of precision that can be established, while trying to navigate various competing experiences and interpretations.

Physicians have the difficult task of trying to overcome, or at least neutralize, the sources of uncertainty that may affect language use. The clinical setting is hardly pristine. Issues arise, for example, related to emotion, memory, past experiences, and personality that compromise clarity. How is certainty possible when language use is influenced by those considerations? What often happens is that attempts are made to marginalize these intervening factors so that the referents of linguistic expressions become more clear. But the clinician, due to the uncertainty associated with language, might have to engage hermeneutics and seek a proper interpretation [22]. Nonetheless, clarity is sought typically in other ways.

### Clarifying language use

The strategies that have been followed to seek clarity are based mostly on empiricism [23]. Specifically, the focus is on collecting objective evidence. The assumption is that misunderstanding can be avoided only if the contingencies that often pervade language use are overcome. An image is created suggesting that language can be stabilized to avoid ambiguity. In the end, regularity must be established.

At this point, a central proposal of Cartesian philosophy, or dualism, comes into play [24]. That is, the attempt is made to strip language of the uncertainty linked to culture, emotion, or personality, for example, that may taint communication in a clinical setting. The aim of this maneuver is to restrict the influence of these and similar interpretive factors, so that assessments are improved. Clinical decisions can thus be made on objective data, as proponents of evidence-based medicine recommend [25].

At this time, medicine is struggling with this issue, with almost an obsession to become evidence-based [26]. In this case, the aim is to achieve the neutrality required to provide objective descriptions of a medical problem. The assumption is that in following the acquisition of objective data correct diagnoses can be made and the proper interventions prescribed. With objective evidence at the foundation, medical decision-making is supposed to become more rational.

Clinicians and researchers have proposed a variety of tactics to achieve this goal. But only three will be discussed at this time. The first is based on the principle of measurement. Through an increased reliance on quantification, some have argued that clinical practices have become more reliable [27]. Numbers, after all, are not thought to be culture-bound and thus should provide a precise description of events. A quick glance at a clinical record reveals a propensity to describe many results in quantitative terms and clearly counting is encouraged by the implementation of electronic patient records [28]. In practice, the language of calculation is concise and readily understandable. Everyone is assumed to know the meaning of a 40% solution or the bio-data derived from a stress test.

The second method relies on standardization and is premised on a basic rule of logic. In this case, clarity is improved when mutually exclusive categories are adopted to classify events and behaviors [29]. With the elimination of any overlap, standardization is possible. In the absence of ambiguity, patient responses should be similar to a particular query and any variation can be assumed to represent these differences. The current proliferation of checklists and pre-programmed, easily processed forms is justified by this principle.

The third tactic is the computerization of clinical practice. Consistent with the idea of technē described by Jacques Ellul, computers are thought to operate according to ineluctable rules [30]. Expert systems, for example, are now available to conduct therapy sessions, organize clinical data, and make diagnoses [31]. The fact that computers are reliable and faithfully follow instructions creates an image of regularity and uniformity. In effect, computers do not tolerate ambiguity, and thus discipline how language is used.

But critics may argue that in a computerized record, space is often allotted for the thinking of patients, or their subjectivity, to be given consideration. In the standardized SOAP format, for example, both the **S**ubjective and **O**bjective features of a problem are documented. Nonetheless, given this dualistic prescription, personal insights are treated mostly as supplementing the objective descriptions, or are downplayed as anecdotal. In either case, clinicians give primacy to what is believed to be objective evidence.

In each tactic, a similar practice is employed to promote clarity--the illusion is created that language use is not situated but universal. Quantification, after all, is touted to be a universal, culture-free language. And because the use of computers epitomizes rationality, and thus is uninfluenced by human foibles, language appears to be severed from everyday speech [32]. Hence the connection between an expression and referent is presumed to be as clean as possible, divorced from alternative uses of language and competing interpretations.

But this search for precision generates an abstract and false image of language. Specifically ignored is how persons, and in this context physicians and patients, use language in everyday life. Joseph Weizenbaum calls this usage “natural language” [33]. The language that is imposed by clinical forms and checklists, for example, is rigid and unrealistic, and thus insensitive to how persons interpret themselves and their situations. Responses may be standardized, and thus precise, but the intentions of patients may be seriously misconstrued. What they have to say is easily concealed behind clear, but irrelevant language.

What is important at this stage in this discussion is whether the indexical thesis adequately characterizes language. Indeed, this theory seems to promote a portrayal that obscures how persons really talk, as a consequence of emphasizing clarity. This thesis, however, has been eclipsed by another that treats language differently. Charles Taylor calls this outlook “expressive-constitutive” [18]. In this relatively new approach, the aim is not to overcome, or neutralize the influence of language, but to appreciate how language use and the worlds of patients are basically connected. This realization, moreover, is purported to enhance clinical practice.

### Language that compels

Because the indexical thesis is dualistic, the opportunity is open for objectivity; that is, subjectivity can be overcome to confront objective facts. In this regard, the descriptive that is used is problematic, that is, a pointer. Language merely indicates something, thereby suggesting that all referents can be examined objectively because their significance is not influenced by subjectivity. A proper investigation, of course, is necessary that relies on empirical data.

Newer theories, however, adopt a different trope, one derived from literature. As Maurice Merleau-Ponty says, language constitutes the “prose of the world” [34]. His point is that as opposed to a pointer, language envelops persons. As a result, language use mediates everything that is known. To paraphrase Roland Barthes, there is no other side to language, where objective connections and referents reside [35]. People are permeated by language; there is no escape. Language cannot be avoided or tamed, so that objectivity is possible.

The unifying feature of new theories of language is a focus on interpretation, which has clear implications for clinical practice. This position is non-dualistic and challenges Cartesianism. Language, in this view, does not merely highlight elements but participates intimately in the identification and arrangement of everything that is known. As suggested by Merleau-Ponty, reality is catalogued through language, including the lives of patients.

This image of language is poetic, but not according to the usual stylistic distinction between poetry and literature. In this context, consistent with Barthes, poetic means that language is creative, evasive, but insightful [36]. Rather than obvious, meanings arise from contrasting interpretations. A patient’s interview or medical record, for example, thus defies formalization and immediate comprehension.

What this shift in thinking means, in clinical practice, is that clarity is not necessarily the gold standard. Because the influence of language can never be cast aside, a new way is needed to think about valid knowledge. In this emerging framework, even attempts to neutralize language merely introduce other modes of talk under the guise of calculation and standardization. But because in those examples language is believed to be neutralized, many erroneous classifications and interpretations go unnoticed.

Instead of seeking clarity, therefore, clinicians have a different responsibility, if one is to take interpretation seriously. With all knowledge mediated by language, clinical practice becomes a hermeneutic activity [37]. Instead of pointing to referents, linguistic expressions convey messages that must be properly deciphered. And rather than clarity, clinicians might focus on making sure they interpret information as patients intend. This outcome, moreover, is not necessarily achieved through precise measurements or classifications. Because the human condition, like prose is fundamentally interpretive, another strategy is required.

This change in orientation, however, does not necessarily mean that medicine should not be evidence-based, although some critics claim that sound practice may be compromised [38]. What passes for evidence, however, must be assessed anew. No longer are objective data, captured through experimental practices, a reliable source of relevant information about a patient or community. In the absence of dualism, evidence has an existential cast, and refers to the personal, and interpersonal, stock of knowledge or interpretations that persons or communities use to define themselves and their situations.

A hermeneutically-based approach recognizes the situatedness of language, since one perspective or another is always revealed [39]. Clarity, therefore, is too limited and sterile. A life that is linguistically mediated is never clearly exposed, but is engaged gradually through a process of give and take that is interrupted by checklists and computer-ready forms [40]. In this environment, a clinician who hopes to communicate effectively with a patient must stop imposing means for achieving clarity and pursue understanding. Medical practice thus has a very special aim--patients must be allowed to speak and be heard in their own voices.

### Looking for the world

As a pointer, language merely indicates something or another. But in interpretive theories of language, expressions are not pointing but are part of a process of creating knowledge. Understanding is achieved, accordingly, not through clarity but a proper interpretation. Rather than attempting to neutralize language, clinicians must recognize the nature of language and attempt to enter the elusive realm of interpretation. In this place, facts are always contested by rival interpretations, instead of objective. Plagued by the creative influence of language, referents of speech are never obvious, but must be disclosed through proper interpretation [41].

Accurate classification and standardization are no guarantee that mutual understanding will be reached. While adhering to what Gabriel Marcel calls the “spirit of abstraction,” both processes rely on the imposition of strategies to foster clarity [42]. But such tactics, rather than culture-free, simply introduce interpretations that may occlude the situational effects of language. True understanding cannot occur through this modus operandi.

Instead of trying to neutralize language, how patients use language must be unpacked [43]. Clinicians, for example, can learn to contextualize patient expressions and treat them as testimonials that should be addressed in the way that they are intended. Accordingly, the intended significance of these meanings can be exposed by clinicians and corroborated through continued and iterative discourse [44]. In fact, only through interpretation is on-going exchange possible that allows a doctor and patient to interact competently. At this point is where the importance of dialogue is revealed.

But dialogue goes beyond merely an exchange of words. As a result, it is important that both a patient and doctor try and enter each other’s worlds. In this regard, the creative nature of language is fundamental, and is the focal point of encountering someone. How persons interpret themselves and others is not something that occurs after their experiences but mediates the process of knowing and interacting. Physician and patient, accordingly, must reflect and wade through a myriad of interpretations to reach a common understanding, which can always be reinterpreted and changed. Dialogue is thus always a fully interpretive and contested exchange.

Again, clarity is not the thrust of dialogue. After all, as suggested above, dialogue is not neat but a process whereby assumptions are challenged until patients are understood in their own terms [39]. A clinician, for example, must reflect on a patient’s expressions, try out an interpretation, and possibly advance another option until some agreement is reached. No outside authority should stifle this search; no objective support can be consulted. Any understanding, accordingly, is always tentative, due to the ambiguity of language. For even agreement involves interpretation, as opposed to a direct encounter with an objective connection or referent.

What clinicians are trying to do, in other words, is enter the linguistically inscribed world of patients. This activity is not as sterile as striving for clarity. There is no attempt to withdraw from or overcome the elusiveness of language, but rather a desire to become immersed in the expressions of patients. A world is announced in these stories that provides important insights into the meaning of health and illness to patients, and how they will respond to these considerations [8].

Focusing on these stories, however, can be problematic [45]. Not everyone tells the truth. Furthermore, some persons are reluctant to talk or exaggerate, and a class bias may be at the root of these differences [46]. But if the conditions for dialogue are created, these issues can be addressed. There is a bigger issue, however, related to power and the resulting structural inequalities [47]. Indeed, persons must have access to treatment before they can tell their stories and be correctly heard. Narrative medicine seems to skirt around this political issue.

But in clinical practice, and community work, dialogue allows language use to be unpacked instead of avoided. What this change signals for clinical practice is that clinicians view themselves as communicating with rather than gathering data about patients. If a device, such as a checklist, violates this principle, other attempts should be made at world entry. Follow-up questions can be asked, along with further discussion to flesh out a response; more intimate consultations should be pursued. Simply following rules, or step-wise instructions, may foster precision but inhibit dialogue. But when the indexical thesis is dismissed, the best alternative is to enter language and the accompanying ambiguity. After all, the significance of patients’ expressions is not found in abstractions but in their worldly significance.

## Conclusion

The main point is that clinicians must listen to their patients [48]. But this message has been given priority in the past. Viewing language differently, however, makes this idea more compelling. With this different portrayal of language in mind, the rationale for listening extends beyond politeness or because patients expect this courtesy. Even a more serious appeal to acquire facts about a patient’s history is not very convincing. But now something more important is revealed by language that compels listening [49]. That is, language use is basic to creating and acquiring valid knowledge about a patient’s medical concerns.

But given the ubiquity of interpretation, equally significant is that the current methods that clinicians use to achieve clarity are outmoded. That is, the emphasis on precision may improve focus and standardization while overlooking a vital element--i.e., the stories that patients are telling. Clarity and regularity may increase the reliability of patient responses, but also could instill a perspective, a linguistic account, that is irrelevant and possibly harmful.

Dialogue, on the other hand, is not pristine but is a valid response to a post-indexical version of language. In this situation, language makes an announcement that should not be ignored by physicians or other providers. Embedded in language is a patient’s world, possibly a very unique reality that holds the key to understanding properly a patient’s symptoms, fears, or interests, all of which are intertwined with their condition and prognosis for returning to health.

Listening, therefore, is not the same as clinging to devices designed to achieve clarity. Nor should listening be equated with acquiring extensive background data. Instead, and most noteworthy, listening consists of following the lead of language, often along many strange paths, until a proper understanding is reached. That is, listening should culminate in world-entry. In this way, a patient’s true background is opened that is required for an effective intervention. The call of language is now truly commanding and far more significant than clarity and objectivity.

## References

[1] Roter DL, Hall JA (2006). Doctors talking with patients/patients talking with doctors: improving communication in medical visits.

[2] Schmidt E, Schopf AC, Farin E (2017). 2017. What is competent communication behaviour of patients in physician consultations? – chronically-ill patients answer in focus groups. Psychol Health Med.

[3] Betancourt JR, Green AR, Carrillo JE. Cultural competence in health care: emerging frameworks and practical approaches. The Commonwealth Fund 2002. http://www.commonwealthfund.org/publications/fund-reports/2002/oct/cultural-competence-in-health-care--emerging-frameworks-and-practical-approaches. Accessed 1 Nov 2017.

[4] Truong M, Paradies Y, Priest N (2014). Interventions to improve cultural competency in healthcare: a systematic review of reviews. BMC Health Serv Res.

[5] Fallowfield L, Guarneri V, Akif Ozturk M, May S, Jenkins V (2014). Blurring of boundaries in the doctor–patient relationship. Lancet Oncol.

[6] Franz B, Murphy JW (2016). Electronic medical records and the technological imperative: the retrieval of dialogue in community-based primary care. Perspect Biol Med.

[7] Waitzkin H (1989). A critical theory of medical discourse: ideology, social control, and the processing of social context in medical encounters. J Health Soc Behav.

[8] Charon R (2008). Narrative medicine: honoring the stories of illness.

[9] Balmer D, Gill A, Nuila R (2016). Integrating narrative medicine into clinical care. Med Educ.

[10] Hutto DD, Brancazio NM, Aubourg J (2017). Narrative practices in medicine and therapy: philosophical reflection. Style: a quarterly journal of aesthetics, poetics, stylistics, and literary. Criticism.

[11] Charon R (2001). Narrative medicine: a model for empathy, reflection, profession, and trust. JAMA.

[12] Marini MG (2016). Narrative medicine: bridging the gap between evidence-based care and medical humanities.

[13] Vannatta S, Vannatta J (2013). Functional realism: a defense of narrative medicine. J Med Philos.

[14] Polkinghorne DE (1988). Narrative knowledge and the human sciences.

[15] Husserl E (1964). The Paris lectures.

[16] Charon R, Wyer P (2008). Narrative and evidence-based medicine. Lancet.

[17] Rozak T (1986). The cult of information.

[18] Taylor C. Heidegger on language. In: Dreyfus HL, Wrathall MA, eds. A Companion to Heidgegger. Oxford: Blackwell; 2005.

[19] Solomon M (2008). (2008). Epistemological reflections on the art of medicine and narrative medicine. Perspect Biol Med.

[20] Morris DB (1991). The culture of pain.

[21] Fischer MA, McKinlay JB, Katz JN, Gerstenberger E, Trachtenberg F, Marceau LD, Welch LD (2017). Physician assessments of drug seeking behavior: a mixed methods study. PLoS One.

[22] Svenaeus F (2001). The hermeneutics of medicine and phenomenology of health: steps toward a philosophy of medical practice.

[23] Rycroft-Malone J, Seers K, Titchen A, Harvey G, Kitson A, McCormack B (2004). What counts as evidence-based practice?. J Adv Nurs.

[24] Bordo S (1987). The flight to objectivity.

[25] Timmermans S, Mauck A (2005). The promises and pitfalls of evidence-based medicine. Health Aff.

[26] Gross R (2001). Decisions and evidence in medical practice: applying evidence-based medicine to clinical decision making.

[27] Dutra L, Campbell L (2004). Quantifying clinical judgment in the assessment of adolescent psychopathology: reliability, validity, and factor structure of the child behavior checklist for clinician report. J Clin Psychol.

[28] Murphy JW, Pardeck JT (1991). The computerization of human service agencies.

[29] Bolter JD (1984). Turing’s man: western culture in the computer age.

[30] Ellul J (1964). The technological society.

[31] Dreyfus HL, Dreyfus SE (1986). Mind over machine.

[32] Winograd T, Flores F (1986). Understanding computers and cognition.

[33] Weizenbaum J (1976). Computer power and human reason.

[34] Merleau-Ponty M (1973). The prose of the world.

[35] Barthes R (1985). The grain of the voice.

[36] Barthes R (1972). Mythologies.

[37] Palmer R (1969). Hermeneutics.

[38] Howick JH (2011). Philosophy of evidence-based medicine.

[39] Gadamer HG (1996). The enigma of health.

[40] Ricoeur P (1991). From text to action: essays in hermeneutics.Volume 2.

[41] Heidegger M (1962). Being and time.

[42] Marcel G (1962). Man against mass Society.

[43] Zaner R (2014). Conversations on the edge.

[44] Madison GB (1981). The phenomenology of Merleau-Ponty: a search for the limits of consciousness.

[45] Kalitzkus V, Matthiessen PF (2009). Narrative-based medicine: Potential, pitfalls, and practice. The Permanente J.

[46] Macnaughton J (2009). The dangerous practice of empathy. Lancet.

[47] Abettan C (2017). From method to hermeneutics: which epistemological framework for narrative medicine. Theor Med Bioeth.

[48] Frank AW (2010). Letting stories breathe.

[49] Zaner R (1981). The context of self: a phenomenological inquiry using medicine as a clue.

